# Impact of an intelligent chronic disease management system on patients with type 2 diabetes mellitus in a Beijing community

**DOI:** 10.1186/s12913-018-3610-z

**Published:** 2018-10-29

**Authors:** Wei Xue-juan, Wu Hao, Ge Cai-ying, Liu Xin-ying, Jia Hong-yan, Wang Li, Guo Xiao-ling, Liu Wan-ying, Gao Wen-juan, Liang Wan-nian

**Affiliations:** 1 Fangzhuang Community Health Service Center of Capital Medical University, Building No.1, 3rd quarter of FangQunYuan, Fangzhuang residence community, Fengtai District, Beijing, China; 2 Health and Family Planning Commission, Badadi, Wenti Rd. No. 2, Fengtai District, Beijing, China; 3 College of General Practice and Continuing Education of Capital Medical University, Xitoutiao No. 10, Youanmenwai, Fengtai District, Beijing, China

**Keywords:** Type 2 diabetes, Intelligent chronic disease management system, Fangzhuang (Beijing), Community

## Abstract

**Background:**

Rapid demographic and economic changes have made chronic disease the number one health issue in China, contributing to more than 80% of the country’s 10.3 million annual deaths and nearly 70% of its total disease burden (Wang et al., Toward a Healthy and Harmonious Life in China: Stemming the Rising Tide of Non-Communicable Diseases, 2011; Yip and Hsiao, Lancet 384: 805-18, 2014). Diabetes is a major contributor to the chronic disease burden and is experienced by nearly 11% of the adult population of China (Yang et al., N Engl J Med 362:1090-101, 2010).

In response to the challenges of chronic disease, the Chinese government initiated comprehensive health care reforms nationwide in 2009. A key measure was a hierarchical diagnosis and treatment system for monitoring and reducing chronic diseases and improving the community health service system (Barber et al., Health Policy Plan 29:367-78, 2014). Primary hospitals, such as community health service centers, are the main gatekeepers for management of diabetes and other chronic diseasesin China. In recognition of the need for a more patient-centered approach, the Chinese government has piloted a program incorporating methods of diabetes self-management for chronic care: the Happy Life Club (Browning et al., Front in Public Health 2:181, 2015). This program is modeled on a similar program developed in Australia (Kelly et al., Aust J Prim Health 9:186-9, 2003). The ICDMS is an important tool in the implementation of patient-centered programs targeting chronic health issues, and its success is determined by factors, such as frequent contact between patients and doctors and effective website training for patients.

This retrospective study used de-identified data from the Fangzhuang (Beijing) intelligent chronic disease management system (ICDMS) database to evaluate the effect of an intelligent chronic disease management system on selected Beijing community patients who have type 2 diabetes mellitus (T2DM).

**Methods:**

A comparative study before and after ICDMS implementation was performed to evaluate the effect of ICDMS on the rates of follow-up and laboratory examinations, measurement rates of blood glucose and lipids, glycosylated hemoglobin (HbA1c) and blood lipid levels, as well as the corresponding health parameters. Continuous variables and categorical variables were analyzed using paired t-test and McNemar’s tests, respectively.

**Results:**

A total of 2451 T2DM patients met inclusion/exclusion criteria. Compared with the pre-index period, the laboratory examination, rates of blood glucose and blood lipids increased significantly in the post-index period (*p* < 0.001). Triglyceride (TC) levels decreased significantly from 5.22 mmol/L to 5.11 mmol/L (*p* < 0.05), and high density lipoprotein-cholesterol (HDL-C) levels increased significantly from 1.35 mmol/L to 1.48 mmol/L (p < 0.05). The control rate of TC increased from 24.86 to 29.76% (*p* = 0.079). The control rate of low density lipoprotein-cholesterol (LDL-C) increased from 12.16 to 13.97% (*p* = 0.421), while the control rate of HDL-C increased significantly from 68.60 to 78.77%. Importantly, Compared with the patients with HbA1C above 7% in the pre-index period, the mean HbA1c decreased significantly from 7.84 to 6.94%((*p* < 0.001) in the post-index period, and the control rate of HbA1c was 57.43%.

**Conclusions:**

The intelligent chronic disease management system is an effective tool in the management of T2DM and should be promoted by the Community Health Service Center in China as well as in other developing countries.

## Background

The rapid development of the economy, the arrival of an aging population, changes in the environment, behavioral lifestyles and other factors have led to a continuous increase in the prevalence and the number of patients of type 2 diabetes [1, 2], which not only seriously has threatened the health of our residents, but also caused a heavy economic burden of disease in China [3]. In response to the challenges of chronic non-communicable diseases represented by type 2 diabetes, the Chinese government initiated hierarchical diagnosis and treatment system reforms nationwide for improving the community health service system in 2009 [4]. In recognition of the need for a more patient-centered approach, the Chinese govenment has piloted a similar program of diabetes chronic self-management care: the Happy Life developed in Australia [5, 6]. The ICDMS is an important tool in the implementation of patient-centered programs targeting chronic health issues.

As a National Demonstration Community Health Service Center in China, the Fangzhuang community health service center in Beijing is one of the sites which first adopted the patient-centered model service. The Fangzhuang community is located in the south of Beijing, and 21.4% of its residents are 60 years of age and older. The Fangzhuang community health service center developed and applied ICDMS to optimize management of chronic diseases [7, 8]. On the basis of the ICDMS clinical application and experience sharing, the standard for internet plus community healthcare management model has been drafted as the standard operating guideline for the ICDMS project [9] and authorized by the Department of National Health and Family Planning Commission of the PRC. In this current study, the effect of the ICDMS on T2DM patients was evaluated in order to provide a reference point for the establishment of community healthcare management and service models in China as well as other developing countries.

## Materials

### Data source

All de-identified data were extracted from the ICDMS, including basic demographics such as gender and age, diagnoses, duration of T2DM and comorbidity conditions, treatments such as annual follow-up times, and laboratory examinations including the tested time and level of blood glucose, HbA1c and lipids (TC, TG, LDL-C, HDL-C). The database included all patients with chronic health conditions managed by the Fangzhuang community health service center. This study was a retrospective analysis of de-identified data, which did not involve collection, use, or transmittal of individually identifiable data; ethics committee approval was not required.

### Design and setting of the study

**Fig. 1:**
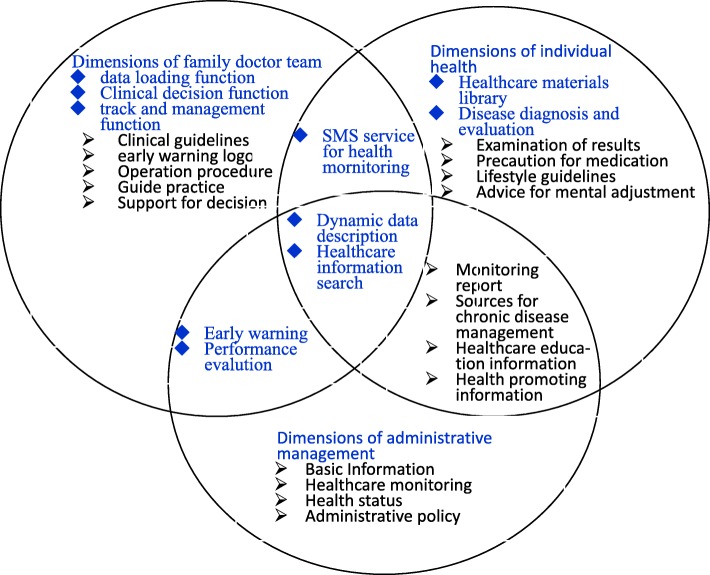
Functions of the intelligent chronic disease management system

Because the ICDMS was developed and officially released on October 31, 2014, the entire study period was set from October 31, 2013 to January 30, 2016. Patients with “diabetes” presented in the text of diagnosis were enrolled. They had been diagnosed as diabetes based on their previous medical history in community health service center. The following study periods were included in this study:

- Pre-index period: a fixed period from October 31, 2013 to October 30, 2014, during which at least one follow-up visit was recorded.
- Index period: a fixed period from November 1, 2014 to January 30, 2015, during which the ICDMS was applied. Multiple functions of the ICDMS were supplemented to share EHRs of all patients among the health service center and tertiary referral hospital, including data sharing, real-time warning, dynamic tracking and continuous data description, data loading and comprehensive analysis, bidirectional referral and performance assessment etc. For example, the present blood glucose level can be displayed automatically and labeled with three colors of icons including red, yellow and blue as the real-time warning signs, which assist family doctors to ensure patient issues related to the management of T2DM can be resolved appropriately and in a timely fashion. At the same time, the solution to the above problems can be supervised by Fangzhuang community administrative department. All the data produced during the patients management process will be auto stored in the ICDMS database (Fig. 1) [8, 9].
- Post-index period: a fixed period from January 31, 2015 to January 30, 2016, during which at least one follow-up visit was recorded.

**Fig. 2:**
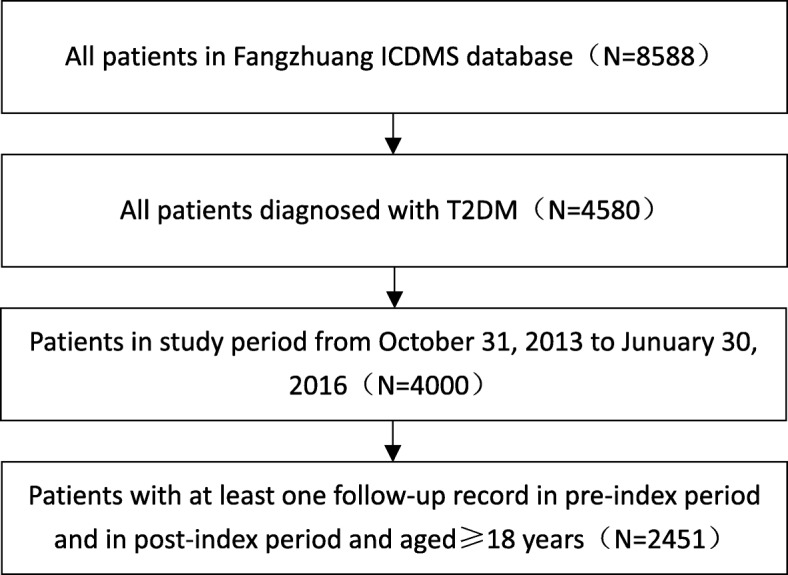
Flow diagram for patient selection

In addition, patients diagnosed with gestational diabetes mellitus, or type I diabetes and younger than 18 years of age were excluded. A total of 2451 cases with T2DM were included in the final analysis (Fig. 2).

### Outcome measures

The outcome measures for this study are defined as follows:

- Demographics and comorbidity conditions. For these measures, the last valid value in the pre-index period was considered as the baseline level.
- Annual follow-up times. The total number of annual follow-up times and the distribution of these follow-up times were calculated in the pre-index and post-index periods.
- Detection rates of blood glucose and lipids. The standard detection rates of blood glucose and blood lipids during the pre-index and post-index periods were calculated based on National Basic Public Health Service Standards [10] and Guidelines for Prevention and Treatment of Type 2 Diabetes in China [11]. According to these standards, fasting blood glucose and postprandial blood glucose should be recorded at each follow-up; glycosylated hemoglobin (HbA1c) should be tested twice at least per year; and blood lipids (total cholesterol [TC], triglyceride[TG], low density lipoprotein cholesterol[LDL-C], high density lipoprotein cholesterol[HDL-C]) should be tested at least once a year. The standard detection rate was calculated by dividing the patient numbers who meet standard detection times requirement by the total number of patients.
- Average change of body weight, HbA1c and blood lipids (TC, TG, LDL-C, HDL-C). The average change during the pre-index and post-index periods were both calculated using the last valid data.
- Change of control rates of HbA1c and blood lipids (TC, TG, LDL-C, HDL-C). For patients with T2DM HbA1c should be controlled to levels below 7% TC should be controlled to levels below 4.5 mmol/L, and TG should be controlled to levels below 1.7 mmol/L [12]. For patients without coronary heart disease (CHD), LDL should be controlled to levels below 2.6 mmol/L. In patients with CHD, LDL should be controlled to levels below 1.8 mmol/L. HDL levels should be targeted to levels higher than 1.0 mmol/L (for males) or 1.3 mmol/L (for females). The control rate was calculated by number of patients reaching control targets by the total number of patients.

### Statistical methods

Descriptive analyses were utilized to describe the characteristics of baseline and each time period. Continuous variables were summarized as means±SD. Categorical variables were reported with frequencies and percentages. Comparisons between the pre-index and post-index periods were made using a paired t-test or Wilcoxon signed-rank test. Categorical variables were compared using McNemar’s test between pre- and post-index periods.*p* < 0.05 was considered to bestatistically significant. The statistical analyses wereperformed using thePython2.7 program and the corresponding statistical package scip-0.17.1 and statsmodels-0.6.1.

## Results

In total, 2451 cases of T2DM patients were recruited, which included 1016(41.45%) males. Twenty-three patients were 18–39 years old (0.94%), 1489patients were 40–64 years old (60.75%), and 939 (38.31%) were over 65 years of age. Patients diagnosed with primary hypertension, dyslipidemia, stroke, chronic heart conditions, mental health problem and cancer accounted for 2337 (95.35%), 819 (33.41%), 93 (3.79%), 1413 (57.65%), 849(34.64) and 5(0.2%) of total cases respectively (Table 1).

**Table 1:** Baseline characteristics of 2451 cases of T2DM patients

General characteristics	Total number, n (%)
Gender	
Male	1016 (41.45)
Female	1435 (58.55)
Age (years)	
18–39	23 (0.94)
40–60	1489 (60.75)
≥ 60	939 (38.31)
Comorbidities	
Hypertension	2337(95.35)
Dyslipidemia	819 (33.41)
Stroke	93 (3.79)
Chronic heart condition	1413 (57.65)
Mental health problem	849(34.64)
Cancer	5 (0.20)

**Fig. 3:**
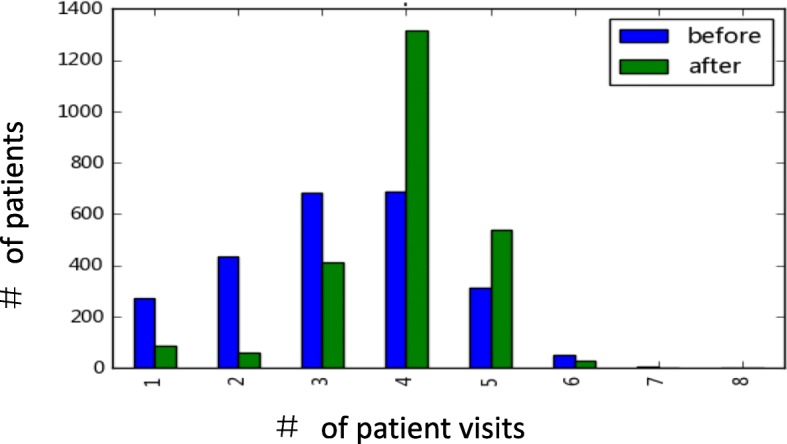
Distribution of follow-up times in the pre-index and post-index periods

A total of 9629 follow-up visits were recorded in the post-index period, representing a significant increase(22.23%) over the pre-index period (7874 follow-up visits; *p* < 0.001). In addition, the average visit number per patient was 3.93 visits in post-index period, which was greater than the 3.21 visits in the pre-index period (p < 0.001); the average follow-up visit time interval was 92.88 days in the post-index period and 113.71 days in pre-index period (*p* < 0.001); A total of 92.5% of patients were followed up regularly (3–5 times per year) in the post-index period, which was greater than 68.7% in the pre-index period (*p* < 0.001, Fig. 3).

Compared with the pre-index period, the standard detection rate for fasting blood glucose (FPG) increased from 18.93 to 64.71%, while the standard detection rate for postprandial blood glucose (PPG) increased from 10.77 to 30.64%. The standard detection rates for HbA1c, TC, TG, LDL-C, and HDL-C all increased, from 51.08 to 69.28%, from 27.01 to 66.83%, from 21.30 to 51.82%, from 27.25 to 63.20%, and from 26.89 to 64.26%, respectively. The standard detection rates for blood glucose and lipids all significantly improved, as well as absolute levels (*p* < 0.001; Table 2).

**Table 2:** Standard detection rate of blood glucose and lipids of 2451T2DM patients

	Pre-index period	Post-index period	*p*-value
	*n* (%)	*n* (%)	
Fasting blood glucose (FPG)	464 (18.93)	1586 (64.71)	< 0.001
Postprandial blood glucose (PPG)	264 (10.77)	751 (30.64)	< 0.001
Glycosylated hemoglobin (HbA1c)	1252 (51.08)	1698 (69.28)	< 0.001
Total cholesterol (TC)	662 (27.01)	1638 (66.83)	< 0.001
Triglyceride(TG)	522 (21.30)	1270 (51.82)	< 0.001
Low density lipoprotein (LDL-C)	668 (27.25)	1549 (63.20)	< 0.001
High density lipoprotein (HDL-C)	659 (26.89)	1575 (64.26)	< 0.001

Compared with the pre-index period, the TC mean significantly declined from 5.22 mmol/L to 5.11 mmol/L (*p* < 0.05); while HDL-C increased significantly from 1.35 mmol/L to 1.48 mmol/L (p < 0.05). The pre-index and post-index means of TG and LDL-C were comparable (Table 3). The mean body weight did not change significantly (66.51 kg in the pre-index period and 66.50 kg in the post-index period). The HbA1c mean increased from 6.09% in the pre-index period to 6.53% in the post-index period. However, Compared with the patients with HbA1C above 7% in the pre-index period, the decrease in the mean HbA1c from 7.84% in the pre-index period to 6.94% in the post-index period was significant (*p* < 0.001).

**Table 3:** Change of blood glucose and blood lipid levels in the pre-index and post-index periods

	*N*	Pre-index period (mean ± SD)	Post-index period (mean ± SD)	Average change^a^ (mean ± SD)	*p*-value
HbA1c (%)	1527	6.09 ± 1.00	6.53 ± 0.83	−0.44 ± 1.13	< 0.001
HbA1c(< 7% in Pre-index period)	1325	5.82 ± 0.63	6.47 ± 0.78	0.65 ± 0.95	< 0.001
HbA1c(≥7% in Pre-index period)	202	7.84 ± 1.19	6.94 ± 1.03	−0.90 ± 1.27	< 0.001
TC (mmol/L)	551	5.22 ± 1.12	5.11 ± 1.11	−0.11 ± 1.12	0.018
TG (mmol/L)	551	1.65 ± 1.14	1.69 ± 1.23	0.04 ± 1.10	0.429
LDL-C (mmol/L)	551	3.15 ± 0.90	3.15 ± 0.91	−0.01 ± 0.94	0.881
HDL-C (mmol/L)	551	1.35 ± 0.30	1.48 ± 0.36	0.13 ± 0.31	< 0.001

^a^Average change calculated by subtracting pre-index from post-index period value

Compared to the pre-index period, the control rate of TC increased from 24.86 to 29.76% in the post-index period, and the control rate of LDL-C increased from 12.16 to 13.97%. The control rate of HDL-C increased significantly from 68.60 to 78.77%. The control rate of HbA1c in the patients with HbA1c less than 7% in pre-index period is 100%, and dropped to 80.91% in post-index period, while in the patients with HbA1c above 7% in pre-index period, the control rate of HbA1c was significantly enhanced to 57.43%in post-indexperiod (*P <* 0.001) (Table 4).

**Table 4:** Control rate of blood glucose and blood lipids of T2DM patients in the pre-index and post-index periods

	N	Pre-index n (%)	Post-index n (%)	*p*-value
HbA1c	1527	1325(86.77)	1188(77.80)	< 0.001
HbA1c (< 7% in Pre-index period)	1325	1325(100)	1072(80.91)	< 0.001
HbA1c(≥7% in Pre-index period)	202	0(0.00)	116(57.43)	< 0.001
TC	551	137 (24.86)	164 (29.76)	0.021
TG	551	373 (67.70)	367 (66.61)	0.659
LDL-C	551	67 (12.16)	77 (13.97)	0.314
HDL-C	551	378 (68.60)	434 (78.77)	< 0.001

## Discussion

Diabetes, hypertension and heart disease are most common and are often comorbid in most community health service centers in China. Our study found that by applying the intelligent chronic disease management system, the standardized management rate of blood glucose and blood lipids, as well as levels of TC, and HDL-C improved significantly, findings which are consistent with previous reports [13–16]. These previous reports suggested that application of such systems improve the clinical outcome of patients withT2DM. This improvement in clinical outcome may be attributed to increased efficiency, enhanced cooperation, and greater interaction between doctors and patients, as well as better performance evaluations [8, 16]. This novel intelligent chronic disease management system is guided by providing real time monitoring and early warning feedback to ensure early diagnosis and early intervention for patients with T2DM. For patients with delayed follow-ups, reminders will be added to patient records for family doctors to proactively contact the patients. This is especially important for patients having a poor understanding of disease control. Such reminders help to maintain continuous health management. A dynamic trend chart combining the scientific and comprehensive evaluation reports and the related health indicators can be generated based on data entered during follow-up of patients. This chart assists family doctors to systematically evaluate the outcome of short-term management, identify health problems in real-time, and accordingly adjust the management protocol. Application of these methods helps to control the blood glucose levels as well as other biochemical parameters in patients with T2DM. In addition, the new intelligent chronic disease management system provides a two-way referral visit, promoting highly efficient clinic visits of patients with T2DM as well as whole-process disease management. Methods involving short text messaging and internet information searches inform the patient and his or her family members of risk factors, thus guiding the self-management of patients with T2DM. Finally, a system of professional performance evaluation of family doctors supports continuous improvement of his or her services, and encourages a more standardized working model of family healthcare, more suited to the practical needs of patients with T2DM. All these functions combine to improve the standardization of team service by the family doctor.

Our results showed that the HbA1c mean increased from 6.09% in the pre-index period to 6.53%in the post-index period. However, compared with the patients with HbA1C above 7% in the pre-index period, the mean HbA1c decreased significantly in the post-index period, the control rate of HbA1c among patients with HbA1C below 7% in the pre-index period was 80.91%, and was 57.3% patients with HbA1C among above 7%. This may be explained by the fact that control rate of HbA1c was already at relatively high levels before intervention, compared with the control rate of blood glucose of T2DM patients at 47.7%, and the comprehensive control rate of blood glucose, blood lipids, and blood pressure at only 5.6% [17]. Moreover, the intelligent chronic disease management system was designed to provide more alerts to the abnormal patients with HbA1C above 7% [8, 9], which maybe make the doctor to care these patients, so these abnormal patients could get better HbA1c outcome. So the system could also improve HbA1c management. However, the system need be optimized to care the normal HbA1c patients to keep them long-term to target.

In this study we also found that, despite widespread availability of Guidelines for Prevention and Treatment of Type 2 Diabetes in China [12] and Guidelines for Prevention and Treatment of Dyslipidemia in Chinese Adults [18], most arteriosclerotic cardiovascular disease(ASCVD) patients with T2DM treated in the Fangzhuang community hospital should be regarded as moderate or high-risk groups for ASCVD and should receive strict hypolipidemic treatment; the control rate for TC is less than 33%, and the control rate of LDL-C is only 13%, which is consistent with findings reported in a recent U.S. study [19]. However, this study reported significantly lower control rates for blood lipids than those reported for patients treated in Chinese hospitals, which was up around 40% [17]. This may be attributed to the fact that management of blood glucose has been assessed as a basic indicator of public health in community medical institutions. However, there has been inadequate attention to lipid management by community healthcare staffs, as well as inadequate education of patients concerning blood lipid control. Therefore, in the future, management and control of blood lipids in patients with T2DM should be strengthened, in order to better prevent cardiovascular complications in diabetic patients.

Real-world data were used in the study to include all patients with T2DM covered by the intelligent chronic system management system in our center, thereby reflecting the actual outcome of this updated system. Objective parameters such as blood glucose and blood lipids were used as outcome measures. These data were imported to the chronic diseases management system directly from the laboratory information system, which ensured the objectivity of data. In addition, within patient comparisons were used in the study to exclude the influence of confounding factors caused by varying characteristics of patients.

Some limitations of the study should be recognized. Firstly, a comparison between patients with T2DM covered in the intelligent chronic disease management system and those not included in the system, and a comparison between Fangzhuang sites with other sites, were not done in a case-control manner, side-by-side. Secondly, the data have not been linked with data from other hospital visits. The observed outcome change may be affected by other hospitals visits. Such data are expected to be considered in future studies, to comprehensively evaluate the performance of the intelligent chronic disease management system. Thirdly, the patients included in the study may be representative of sicker patients with more comorbid conditions such as primary hypertension, chronic heart conditions and mental health problem, and may be representative of many urban patients in community health service centers in China, who are elderly and often with more comorbid chronic conditions, and are willing to visit the community health service center where they doesn’t need take much time in hospital visit. Further studies of less sick patients are needed. In addition, the hemoglobin a1c threshold used is 7%, without analyzing variable goals based on the patient’s age, comorbidities, complications, duration of T2DM etc. because the one goal is very easy to implement in the community hospital. Finally, because of the short operating time of the system, long-term effects on clinical end points (such as cardiovascular and cerebrovascular events) have not yet been analyzed and await future study.

## Conclusions

The intelligent chronic disease management system provides a scientifically-based and efficient technology platform, providing key functions for multiple links to optimize management of patients with T2DM. In this current study, the system effectively improved the behavior of primary care providers to manage patients with T2DM with better clinical outcomes.

## Abbreviations

ASCVD: Arteriosclerotic cardiovascular disease
FPG: Fasting blood glucose
ICDMS: Intelligent chronic disease management system
LDL-C: Low density 222 lipoprotein cholesterol
PPG: Postprandial blood glucose
PRC: People’s Republic of China
T2DM: Type 2 diabetes mellitus
TC: Total cholesterol
TG: Triglyceride
